# Detection of extracellular vesicles in plasma and urine of prostate cancer patients by flow cytometry and surface plasmon resonance imaging

**DOI:** 10.1371/journal.pone.0233443

**Published:** 2020-06-04

**Authors:** Linda G. Rikkert, Leonie de Rond, Annemieke van Dam, Ton G. van Leeuwen, Frank A. W. Coumans, Theo M. de Reijke, Leon W. M. M. Terstappen, Rienk Nieuwland

**Affiliations:** 1 Department of Medical Cell BioPhysics, University of Twente, Enschede, The Netherlands; 2 Laboratory of Experimental Clinical Chemistry, Amsterdam UMC, University of Amsterdam, Amsterdam, The Netherlands; 3 Vesicle Observation Center, Amsterdam UMC, University of Amsterdam, Amsterdam, The Netherlands; 4 Biomedical Engineering and Physics, Amsterdam UMC, University of Amsterdam, Amsterdam, The Netherlands; 5 Department of Urology, Amsterdam UMC, University of Amsterdam, Amsterdam, The Netherlands; The Ohio State University, UNITED STATES

## Abstract

Large (> 1 μm) tumor-derived extracellular vesicles (tdEVs) enriched from the cell fraction of centrifuged whole blood are prognostic in metastatic castration-resistant prostate cancer (mCRPC) patients. However, the highest concentration of tdEVs is expected in the cell-free plasma fraction. In this pilot study, we determine whether mCRPC patients can be discriminated from healthy controls based on detection of tdEVs (< 1μm, EpCAM^+^) and/or other EVs, in cell-free plasma and/or urine. The presence of marker+ EVs in plasma and urine samples from mCRPC patients (n = 5) and healthy controls (n = 5) was determined by flow cytometry (FCM) and surface plasmon resonance imaging (SPRi) using an antibody panel and lactadherin. For FCM, the concentrations of marker positive (^+^) particles and EVs (refractive index <1.42) were determined. Only the lactadherin^+^ particle and EV concentration in plasma measured by FCM differed significantly between patients and controls (p = 0.017). All other markers did not result in signals exceeding the background on both FCM and SPRi, or did not differ significantly between patients and controls. In conclusion, no difference was found between patients and controls based on the detection of tdEVs. For FCM, the measured sample volumes are too small to detect tdEVs. For SPRi, the concentration of tdEVs is probably too low to be detected. Thus, to detect tdEVs in cell-free plasma and/or urine, EV enrichment and/or concentration is required. Furthermore, we recommend testing other markers and/or a combination of markers to discriminate mCRPC patients from healthy controls.

## Introduction

Circulating tumor cells (CTCs) and large (> 1 μm) tumor-derived extracellular vesicles (tdEVs) measured with the CellSearch are both associated with poor survival of metastatic castration-resistant prostate cancer (mCRPC) patients [1–4]. Furthermore, changes in concentrations of CTCs and tdEVs after initiation of therapy can be used to monitor treatment response. CellSearch identifies CTCs and large tdEVs in the *cell fraction* of centrifuged whole blood by using EpCAM immunomagnetic enrichment combined with immunofluorescence labeling, and defines both by cytokeratin expression and lacking the leukocyte marker CD45. Discrimination between CTCs and tdEVs is obtained by cell morphology features, and the presence of a nucleus in case of CTCs. For mCRPC patients, a median of ~7 CTCs and ~116 large tdEVs per 7.5 mL of blood was reported (i.e. ~0.009 CTCs and ~0.015 large tdEVs per μL) [1]. As the EV concentration increases with decreasing size [5], a 100–1,000 fold higher concentration of small (< 1 μm) tdEVs is expected in blood of mCRPC patients, resulting in an expected concentration of ~1.5–15 small tdEVs per uL blood. The majority of small tdEVs should be present in *cell-free* plasma of mCRPC patients [6], and may directly be measured in plasma without enrichment techniques. In this study, we performed a pilot study to determine whether mCRPC patients can be discriminated from healthy controls based on the presence of small tdEVs (< 1 μm, EpCAM^+^) and other EV subtypes, directly in cell-free plasma and/or urine. Since EVs <1 μm are below the detection limit of the CellSearch Analyzer, we used flow cytometry (FCM) and surface plasmon resonance imaging (SPRi) in this study.

## Methods

### Minimal detectable marker^+^ particle concentration for FCM

To determine the minimal detectable marker^+^ particle concentration for FCM, we performed a spiking experiment (S1 File). Conditioned medium containing Cell-derived EVs (PC3 cells), identified by CD63-PE, was mixed with plasma at different volumetric dilutions. The minimal detectable marker^+^ particle concentration was defined as the concentration at which the number of detected CD63^+^ particles exceeded the 95% confidence interval based on pure plasma.

### Sample collection

Blood and urine were obtained from five mCRPC patients and five healthy male controls. See Table 1 for donor characteristics. Samples were collected with written informed consent in accordance with the Helsinki Declaration and approved by the medical-ethical assessment committee of the Academic Medical Center, University of Amsterdam (NL64623.018.18). For each donor, whole blood was collected using a 21G needle, and the first vacutainer was discarded. Next, three 2.7 mL citrate vacutainers (BD Biosciences, San Jose, CA) were collected, mixed by gentle inversion and processed within 20 minutes. Vacutainers were centrifuged at 2,500 *g*, 20°C for 15 minutes without brake in a Rotina 380R centrifuge (Hettich, Tuttlingen, Germany). Plasma was collected until approximately 0.5 cm above the pellet using a 5 mL Pasteur pipet (VWR, Radnor, PA). The plasma was pooled and transferred to a conical base tube (10 mL; Sarstedt, Nümbrecht, Germany), and centrifuged at 2,500 *g*, 20°C for 15 minutes. The supernatant was transferred to a conical base tube and divided over 75 μL cryovials (Sarstedt), followed by snap-freezing in liquid nitrogen and storage at -80°C.

**Table 1:** Metastasized castration-resistant prostate cancer (mCRPC) patients and healthy controls characteristics.

Variable	mCRPC patients (n = 5)	Healthy controls (n = 5)
Age in years, mean (range)	80 (66–83)	28 (19–40)
Ethnicity	Caucasian	Caucasian
Mean (range) serum PSA level (ng/mL)*	6.0 (0.1–17.6)	NA
Mean (range) total Gleason score (biopsy tissue)	7.8 (6–9)	NA
Previous treatment for prostate cancer**	Yes	NA

mCRPC: metastasized castration-resistant prostate cancer; NA: not applicable; PSA: prostate specific antigen

*last determined PSA level before inclusion

**including surgery, hormone therapy, radiotherapy, cryotherapy, and/or chemotherapy.

First catch-urine (no prostate massage) was collected in 125 mL routine urine collection cups free of any preservatives. Urine samples were placed on melting ice (0°C) and transported to the laboratory within one hour after collection. The urine collection cup was inverted five times to resuspend the cells. To remove the cells, two aliquots of 20 mL urine were centrifuged at 500 *g*, 4°C for 20 minutes without a brake using a Rotina 46RS centrifuge (Hettich). The supernatant was pooled, vortexed for at least 10 s and aliquots of 1 mL were pipetted in cryovials (Sarstedt), followed by snap-freezing in liquid nitrogen and stored at -80°C.

### Sample preparation

All samples were thawed in a 37°C water bath. For the FCM measurements, the urine samples showed a high level of autofluorescence in the FITC channel, which disappeared after size exclusion chromatography (SEC). Therefore, for each urine sample, 150 μL of urine was loaded on SEC columns (qEVsingle/70 nm; Izon Science, Christchurch, New Zealand), followed by elution with phosphate buffered saline (PBS; 21-031-CV; Corning, Corning, NY). The first 1250 μL eluate was discarded, and the next 500 μL eluate, containing particles with a diameter larger than 70 nm, was used for the FCM measurements. In preliminary experiments, undiluted urine samples caused negative SPRi responses, which disappeared after 5-fold dilution in PBS. Therefore, all samples were pre-diluted 5-fold in PBS for SPRi.

### Markers

A panel of fourteen markers was selected. We included lactadherin, CD9, CD63 and CD81 which are associated with EVs [7, 8], CD45 expressed by leukocytes, CD61 expressed by platelets, CD146 expressed by endothelial cells and CD235a expressed by erythrocytes. In addition, we included EpCAM expressed on cells of epithelial cell origin [1], PSMA expressed on prostate cells [9] or markers reported to be overexpressed on cancer cells CD24, CD47, CD142, HER2 [10–13], and an isotype control. An overview is shown in Table 2.

**Table 2:** Markers used for flow cytometry (FCM) and surface plasmon resonance imaging (SPRi).

Marker	Target protein	Clone	Concentration (μg/mL)		Conjugate	Company
			FCM	SPRi		
**CD9**	Tetraspanin	M-L13	2.5	5	PE	BD Biosciences, San Jose, CA
**CD24**	Sialoglycoprotein	SN3	Unknown	5	PE	Thermo Fisher Scientific, Waltham, MA^1^; eBioscience, San Diego, CA^2^
**CD45**	Protein tyrosine phosphatase, receptor type C	HI30	50	5	FITC	Biolegend, San Diego, CA^1^; Sony Biotechnology, San Jose, CA^2^
**CD47**	Integrin associated protein	CC2C6	8	5	PE	Biolegend, San Diego, CA
**CD61**	Integrine beta-3	VI-PL2	3	5	PE	Thermo Fisher Scientific, Waltham, MA^1^; BD Biosciences, San Jose, CA^2^
**CD63**	Tetraspanin	H5C6	6.25	5	PE	BD Biosciences, San Jose, CA^1^; Cell Guidance Systems, Cambridge, UK^2^
**CD81**	Tetraspanin	JS-81	5	5	PE	BD Biosciences, San Jose, CA
**CD235a**	Glycophorin A	JC159	100	5	PE	Agilent Technologies, Santa Clara, CA^3^
**CD142**	Tissue factor	HTF-1	9	5	PE	BD Biosciences, San Jose, CA^1^; eBioscience, San Diego, CA^2^
**CD146**	Melanoma cell adhesion molecule	P1H12	12.5	5	FITC	Sony Biotechnology, San Jose, CA^1^; eBioscience, San Diego, CA^2^
**EpCAM**	Epithelial cell adhesion molecule	VU1D9	6	5	PE	Thermo Fisher Scientific, Waltham, MA^1^; Own hybridoma cell line, Enschede, Netherlands^3^
**HER2**	Erb-b2 receptor tyrosine kinase 2	24D2	25	5	PE	Biolegend, San Diego, CA
**Lactadherin**	Milk fat globule-EGF factor 8	NA	41.5	5	FITC	Haematologic Technologies, Essex Junction, VT^3^
**PSMA**	Glutamate carboxypeptidase II	REA408	11	5	PE	Miltenyi Biotec, Bergisch Gladback, Germany
**IgG1**	Immunoglobuline G	X40	50	5	FITC/PE	BD Biosciences, San Jose, CA

FITC: fluorescein isothiocyanate; NA: not applicable; PE: phycoerythrin

^1^FCM

^2^SPRi

^3^Same conjugated antibody used for FCM and SPRi.

In FCM, the PE-conjugated isotype control resulted in PE positive concentrations higher than the PE positive concentration found for the actual markers. The PE-conjugated isotype control was considered unreliable, and measurements of marker in PBS were used to estimate the background.

Samples were measured simultaneously by FCM and SPRi, alternating between samples from patients and controls, followed by PBS samples.

### Flow cytometry

Plasma samples were pre-diluted in PBS to event rates between 2,000 and 3,000/s to prevent swarm detection when triggering on side scatter [14]. The pre-dilution varied per plasma sample, and ranged between 10-fold to 100-fold dilution. The urine samples were diluted by SEC, and no additional dilution was necessary to prevent swarm detection. Antibody aggregates were removed by centrifugation at 18,890 *g*, 20°C for 5 minutes prior to use. 20 μL of pre-diluted sample was incubated with 2.5 μL marker (for concentrations see Table 2) for two hours at room temperature in the dark. The staining was stopped by adding 200 μL PBS. All samples were analyzed on an A60-Micro (Apogee; Northwood, UK) at a flow rate of 3.0 μL/minute. Each sample was measured for four minutes or until 500,000 events were detected, triggering on 405-nm side scatter using a threshold corresponding to a side scattering cross section of 10 nm^2^ (Rosetta Calibration; Exometry, Amsterdam, The Netherlands). Taking into account the sample dilution and measurement time, a total of approximately 0.008 to 0.08 μL for plasma and 1 μL SEC eluate of urine was analyzed.

Data analyses were performed in MATLAB R2018b (Mathworks, Natick, MA) and FlowJo (v10.6.1; FlowJo, Ashland, OR). First, for samples with a minimum count rate of 250 counts/s, time points of the data in which the count rate deviated >25% from the median count rate were removed to account for fluctuations in the flow rate. Next, fluorescent gates were determined for every marker, resulting in the gates as mentioned in the supplemented MIFlowCyt in S1 Appendix. Positive (^+^) events are defined as events with a fluorescent signal exceeding the gate. Using this conventional gating strategy, all marker^+^ particles are taken into account, including non-specific binding of markers to lipoproteins or protein aggregates. Recently, we showed that marker^+^ EVs can be distinguished from other marker^+^ particles using the refractive index (RI) [15]. Therefore, we also determined the RI of all particles > 200 nm using Flow-SR as described previously [15]. Briefly, using the ratio of side versus forward scatter, Mie theory, and a mathematical model of the optical configuration of the flow cytometer, the diameter of each particle can be determined. The RI can subsequently be derived from a lookup table of side scatter versus diameter. In the present study, marker^+^ particles include all particles above the size detection limit (Table 3) and positive for a marker, whereas marker^+^ EVs include particles > 200 nm with an RI < 1.42 and positive for a marker.

**Table 3:** Characteristics of the methods used for the detection of extracellular vesicles (EVs).

	Flow cytometry	SPRi
**Detection of**	Single EVs	Ensemble EVs
**EV size detection limit**	≥160 nm*	All EVs
**Particle detection**	In suspension	On surface
**Signal caused by**	Light scattering, fluorescence	Refractive index change
**Minimal # EVs/μL**	1x10^3^**	2x10^5^ [16]
**Antigen detection limit**	70 molecules (PE)	1
**Preparation steps**	Sample dilution, fluorescent marker staining	Sample dilution, printing markers on sensor
**Preparation time**	2.5 hours	2 hours
**Measurement time / sample**	1–4 minutes***	1 hour
**Output**	Antigen expression, concentration, size, refractive index	Antigen expression

* As determined using Rosetta Calibration (Exometry, Amsterdam, The Netherlands) using a core-shell model with refractive index (RI) of 1.38 for the core, 1.48 for the shell and a shell thickness of 4 mm.

** Based on the 95% confidence interval for buffer only measurements (n = 3).

*** Increases with the number of markers to be analyzed.

Concentrations provided are the number of detected events corrected for the total sample dilution, flow rate and measurement time. Because for urine, the dilution caused by SEC was unknown, the marker^+^ concentration is expressed as marker^+^ particles/EVs per μL SEC eluate. For a detailed description of the flow cytometer configuration, operating conditions and data analysis please see the supplemented MIFlowCyt list in S1 Appendix.

### Surface plasmon resonance imaging

SPRi measurements were performed using the MX96 SPRi device (IBIS technologies B.V., Enschede, the Netherlands) as described by Stravers et al. [17]. Markers (5 μg/mL) were printed in triplicate on a sensor surface (Easy2Spot type-G; Ssens B.V., Enschede, The Netherlands) using a Continuous Flow Micro-spotter (CFM) 2.0 (Wasatch microfluidics LLC, Salt Lake City, UT). Three spots were used as control spots, and active sites on the sensor surface were inactivated [17]. In total four sensors were used, each analyzing six samples, i.e. five plasma or urine samples and one negative control (PBS).

Before starting the six sample runs, three regeneration runs were applied, consisting of i) a baseline phase of two minutes, ii) an association phase of 15 minutes, whereby regeneration buffer (0.1 M Glycine (Merck, Darmstad, Germany), pH 3.06 and 0.3% (v/v) Triton-x100 (Sigma-Aldrich, Darmstadt, Germany)) is flowed back and forth over the sensor surface, and iii) a dissociation phase of two minutes, whereby the regeneration buffer is replaced by PBS. Next, 300 μL of each pre-diluted sample was placed into the SPRi to allow measurement of each sample in duplicate. A single sample run consisted of i) a baseline phase of two minutes, ii) an association phase of 60 minutes, whereby the sample is flowed back and forth over the sensor surface and iii) a dissociation phase of four minutes. After each sample run, the chip surface was regenerated to break the formed antigen-antibody bonds before the next sample run was started. Taking into account the sample dilution, 26 μL of plasma and urine was analyzed per sample run.

File conversion and analysis was performed as described by Stravers et al. [17]. SPRi signals in PBS were set to zero to account for differences in the initial spot values. The first 100 s of the association phase was removed from the analysis to account for RI differences of buffers. The mean SPRi signals of each marker of the last 50 s of the association phase, corrected for the reference spot, was used as the maximum response. The difference between the maximum response and the response after 100 s of each spot was used to calculate the mean response in resonance units (RU). While three spots were printed for each marker, we included data from the two spots with the highest relative ligand density (RLL) because of printing artifacts in some spots. A data point represents the RI change during one hour per sample averaged over the two spots. To correct for degeneration of the sensor and for differences in the total particle concentration between samples, the response per sample was normalized by dividing the response for one marker by the sum of all marker responses for that sample. The mean of the duplicates was calculated to obtain a response per marker per donor.

### Data analysis

Median and standard deviation (SD) of the data was calculated per marker. The median is preferred over the mean, because the median is less influenced by outliers and therefore more representative given the small number of samples. Statistical analysis was performed using a Student’s t-test. All analyses were performed in MATLAB R2018b (Mathworks, Natick, MA).

## Results

### Minimal detectable marker^+^ particle concentration for FCM

**Fig 1:**
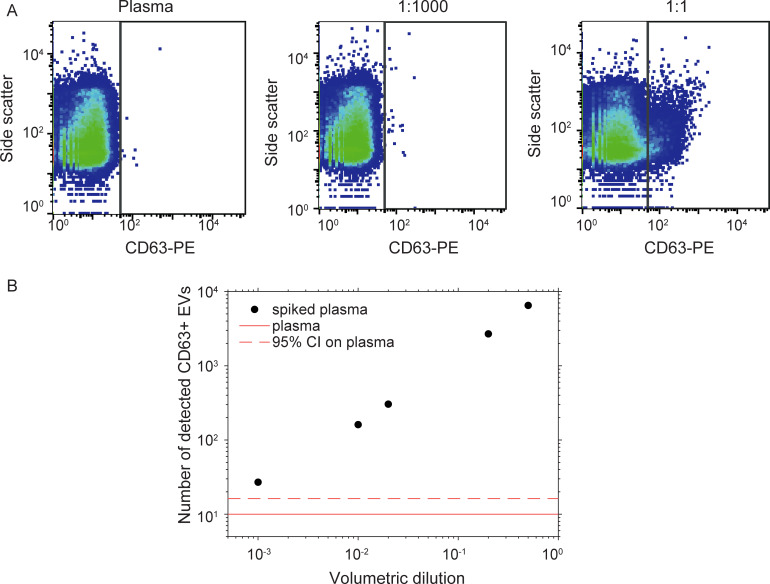
Spiking experiment to determine the minimal detectable marker+ particle concentration for flow cytometry. A) Side scatter versus CD63-PE fluorescence for pure plasma (left), plasma spiked with PC3 extracellular vesicles (EVs) at a 1:1000 volumetric dilution (centre) and 1:1 volumetric dilution (right). B) Detected number of CD63^+^ particles (black dots) at volumetric dilutions of PC3 EVs in plasma. CI: confidence interval.

Fig 1A shows the side scatter versus CD63-PE fluorescence scatter plots for pure plasma, and for PC3 EVs spiked in plasma at a 1:1,000 and 1:1 volumetric dilution. Fig 1B shows that at a volumetric dilution of 1:1,000, the number of detected CD63^+^ particles exceeded the 95% confidence interval for pure plasma. Correction for the particle concentration of the PC3 EV sample and the percentage of CD63^+^ particles in the sample, led to a minimal detectable concentration of 15 marker^+^ particles/μL. Since the A60-micro FCM used in this study has a higher scatter sensitivity and similar fluorescence sensitivity than the A50-micro used in the spiking experiment, we expect the minimal detectable concentration for the A60-micro to be similar or lower than 15 marker^+^ particles/μL.

### Flow cytometry

**Fig 2:**
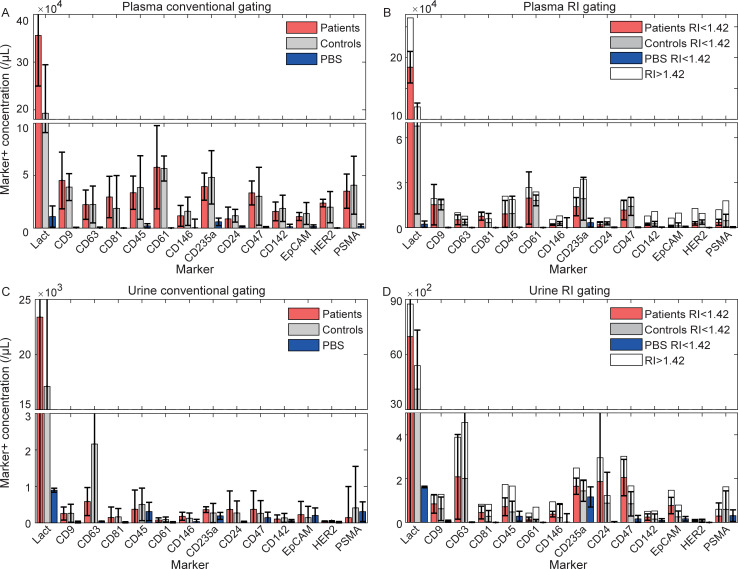
Marker^+^ concentration by flow cytometry per μL plasma (A-B) or urine (C-D) from metastatic castration-resistant prostate cancer patients (red, n = 5), healthy controls (grey, n = 5) and phosphate buffered saline (blue, n = 2). Panels B and D show the refractive index (RI) of marker+ particles >200 nm. Data shown represent the median and standard deviation (whiskers) per group. Lact: lactadherin.

Fig 2 shows the marker^+^ concentration measured by FCM in plasma (Fig 2A and 2B) and urine (Fig 2C and 2D) of patients and controls, and in PBS. Fig 2A and 2C show the marker^+^ particle concentration obtained using conventional gating, that is including all marker^+^ particles. Fig 2A shows that for all markers studied, the marker^+^ particle concentrations exceeded the background measured in PBS (p<0.05). However, only the lactadherin^+^ particle concentrations differed significantly between patients and controls (p = 0.042). The EpCAM^+^ and PSMA^+^ particle concentrations were similar in plasma samples from patients and controls, making it unlikely we detected tdEVs.

Fig 2B shows the RI of marker^+^ particles >200 nm in plasma of patients and controls, and in PBS. When using the RI to identify true marker^+^ EVs (RI<1.42) in the sample, marker^+^ EVs were found for all markers except for CD146, and EpCAM in controls, which did not exceed the background measured in PBS (p>0.05). EpCAM and PSMA mainly stained particles with an RI>1.42, which most likely are lipoproteins [15]. Again, only lactadherin showed a significant difference in the marker^+^ EV concentration between patients and controls (p = 0.017).

Marker^+^ concentrations in urine (Fig 2C) were approximately 10-fold lower than marker^+^ concentrations in plasma. Marker^+^ particles were found for lactadherin, CD9, CD63, CD81, CD61, CD146, CD235a in patients, CD24, CD47 in patients, CD142, and HER2 in patients (p<0.05). However, neither of these marker^+^ concentrations differed significantly between patients and controls. When using the RI to identify true marker^+^ EVs in the sample, concentrations exceeding the background in PBS were found for all markers except for CD146, CD235a in controls, CD142 in controls, EpCAM in controls, and PSMA in patients (p>0.05). However, again none of the markers exceeding the background showed a significant difference in the marker^+^ EV concentration between patients and controls.

Taken together, the lactadherin^+^ particle and EV concentration in plasma differed significantly between patients and controls as measured by FCM. All other markers tested on plasma and urine samples that showed concentrations exceeding the background, did not show a significant difference between patients and controls.

### Surface plasmon resonance imaging

**Fig 3:**
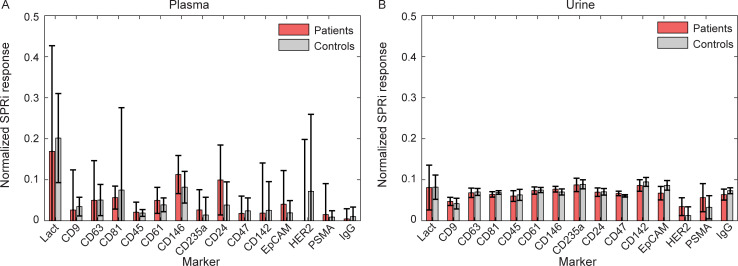
Surface plasmon resonance imaging (SPRi) response for plasma (A) or urine (B) of metastatic castration-resistant prostate cancer patients (red, n = 5), healthy controls (grey, n = 5) and phosphate buffered saline (blue, n = 2) for different markers. Data shown represent the median and standard deviation (whiskers) per group. Lact: lactadherin; RU: resonance unit.

Fig 3A shows the normalized SPRi responses per marker as measured for plasma from patients and controls. Particles expressing lactadherin, CD63, CD81 in patients, CD61 in patients, CD146, CD24 in patients and CD47 in patients were found in plasma, since their responses exceeded the IgG response (p<0.05). However, none of these marker responses differed significantly between plasma of patients and controls.

Fig 3B shows the normalized SPRi responses per marker as measured for urine from patients and controls. Particles expressing CD235a in controls, CD47 in controls and CD142 were found, since their responses exceeded the IgG response (p<0.05). Similar as in plasma, none of these markers differed significantly between plasma of patients and controls.

## Discussion

We determined whether mCRPC patients could be discriminated from healthy controls based on the presence of EV subtypes in plasma and/or urine samples, using FCM and SPRi. FCM was chosen because FCM is frequently used to measure the concentration of EVs in clinical samples such as plasma or urine. However, the majority of common flow cytometers do not detect EVs smaller than 300 nm [18]. For example, the FCM used in this study has an EV size detection limit of 160 nm and an antigen detection limit of 70 PE molecules (Table 3). In SPRi, multiple EVs captured on a surface coated with markers induce a change in the index of refraction, thereby generating the SPRi signal. All EVs, also the EVs too small to be detected by FCM, may contribute to the SPRi signal. However, since an ensemble of EVs binds to the surface, evaluation of the concentration of EVs is not possible with SPRi [16].

Only for the lactadherin^+^ particle and EV concentration in plasma measured by FCM, a significant difference was found between patients and controls. Use of the RI to identify true marker^+^ EVs increased the specificity by exclusion of marker^+^ lipoproteins (RI>1.42) [15]. The cause of the significant difference in lactadherin^+^ particle and EV concentration may be due to the presence of prostate cancer, but the influence of the age [19] and treatment differences between the groups cannot be excluded (Table 1). All other markers did not result in signals exceeding the background in PBS, or showed no significant differences between patients and controls.

Various explanations for the absence of a significant difference in small tdEVs between patients and controls are conceivable. First, no detectable differences are present in EV subtypes <1 μm in the plasma and/or urine samples of the two groups. The small EVs may be bound to or taken up by leukocytes or endothelial cells, and thus will be absent in the cell-free plasma after centrifugation [20]. Furthermore, target proteins, like EpCAM, may be cleaved from EVs, which may restrict the capture and/or detection EVs [21].

Secondly, extrapolation of the concentration of CTCs and large tdEVs found in a previous study [1], results in an expected small tdEV (< 1 μm, EpCAM^+^) concentration of 1.5-15/μL in plasma [22]. Based on our spiking experiment, we expect the minimal detectable concentration for the A60-micro to be 15 marker^+^ particles/μL. However, since we measured plasma volumes < 0.1 μL, it was impossible to detect the few tdEVs that might be present in the plasma sample. For SPRi, a minimum concentration of 10^5^ marker^+^ particles/μL is needed to obtain a signal (Table 3). Therefore, it is likely that the concentration of tdEVs is too low to be detected with SPRi.

A third explanation is that the actual small tdEV concentration is less than the expected small tdEV concentration based on the extrapolation of CTC and large tdEV concentrations [22]. For example, the detected concentrations of small (here 160–1,000 nm) leukocyte EVs was 9.3 x 10^3^ per μL plasma in controls. The concentration of large (> 1 μm) leukocyte EVs was found to be ~900 per μL blood in controls [23]. Thus, the concentration of small leukocyte EVs was not, as mentioned in the introduction, 100–1,000 fold higher than the concentration of large leukocyte EVs, but only 10-fold higher.

Another explanation is that the antigen density on small EV subtypes may be below the antigen detection limit for FCM. For example, the detection limit for EpCAM-PE used in this study was 70 mean equivalent soluble fluorophore (MESF; Table 3 and supplemented MIFlowCyt in S1 Appendix). Since the dye to protein ratio of PE-conjugated antibodies is typically 1, this means that an EV should have a minimum of 70 EpCAM molecules for it to be detected as EpCAM^+^, assuming the staining efficiency is 100%. If we extrapolate the measured EpCAM density on CTCs described by Rao et al. [24] to EVs with a diameter of 300 nm, the expected number of EpCAM molecules is ~40, which is below the detection limit for EpCAM in this study.

Biggs et al. [25], showed a difference in the detection of PSMA^+^ particles between controls and prostate cancer patients with a Gleason score higher than 8. However, the PSMA clone (3/E7) used by this and one other study [25, 26] differs from the one used in our study (REA408), and is not commercially available. Logozzi et al. [27] detected a higher concentration of EVs expressing CD81 and PSA in blood from prostate cancer patients compared to controls. However, in that study the EVs were concentrated by ultracentrifugation before measurements. In our study, the goal was to detect EVs directly in plasma and urine and therefore we did not concentrate the EVs before measurements.

To investigate whether small tdEVs are present in the plasma and/or urine of patients, we recommend to perform an enrichment or concentration enhancement step to increase the concentration of small tdEVs. Furthermore, other clones/markers and/or a combination of markers may be needed, as used by Nanou et al. [1]. Moreover, for FCM, the development of brighter fluorophores and/or more sensitive fluorescence detectors is required to allow detection of the low number of antigens present on EVs. For SPRi, EV concentrations need to be increased to reach the minimal detectable concentration, and/or the SPRi response per EV needs to be increased. Furthermore, SPRi has not often been applied to plasma samples, therefore further optimization may be needed to prevent non-specific binding of for example (lipo)proteins.

In conclusion, only the lactadherin^+^ particle and EV concentration measured in plasma by FCM differed significantly between patients and controls. Based on this result, FCM may be more promising for application to clinical samples compared to SPRi, taken into account the before mentioned challenges. In the future, larger cohort studies are needed to confirm the presence of small tdEVs in blood plasma and urine, and to see whether such EVs can be used to discriminate between prostate cancer patients and controls.

## Supporting information

S1 File Protocol spiking experiment. (DOCX)

S1 Appendix (DOC)

## Abbreviations

CTCs: Circulating tumor cells
EVs: Extracellular vesicles
FCM: Flow cytometry
mCRPC: Metastasized castration-resistant prostate cancer
PBS: Phosphate buffered saline
SPRi: Surface plasmon resonance imaging
tdEVs: Tumor-derived extracellular vesicles
