# Supplementary material for: Detection of extracellular vesicles in plasma and urine of prostate cancer patients by flow cytometry and surface plasmon resonance imaging
Source: PLoS One. 2020 Jun 4;15(6):e0233443. doi: 10.1371/journal.pone.0233443 (PMC7272016; doi:10.1371/journal.pone.0233443)
Supplement: S1 File — (DOCX) [file pone.0233443.s001.docx]

**PC3 EVs**

The prostate cancer cell line PC3 was purchased from the American Type Culture Collection (ATCC, Manassas, VA) and used to produce prostate cancer-derived EVs. The cell line was cultured at 37 °C and 5% CO_2_ in RPMI-1640 with L-glutamine medium (Lonza, Basel, Switzerland) supplemented with 10% v/v fetal bovine serum (FBS), 10 units/mL penicillin, and 10 μg/mL streptomycin. Medium was refreshed every second day. The initial cell density was 10,000 cells/cm^2^ as recommended by the ATCC. When cells reached 80−90% confluence, they were washed three times with PBS and FBS-free RPMI medium supplemented with 1 unit/mL penicillin and 1 μg/mL streptomycin was added to the cells. After 48 h of cell culture, cell supernatant was collected and centrifuged for 30 minutes at 1,000 *g*. The supernatant was pooled, and aliquots of 50 μL were frozen in liquid nitrogen and stored at −80 °C.

**Plasma**

Blood was obtained from four healthy and overnight fasting male donors with written informed consent in accordance with the Declaration of Helsinki. The study protocol was waived by the medical ethics committee of the Amsterdam UMC. Whole blood was drawn using a 21G needle. The first 3.5 mL was discarded. In total 27 citrate vacutainers of 9 mL (BD, San Jose, Ca) were collected, mixed gently with the coagulant and processed within 15 minutes after collection. Plasma was obtained from whole blood by double centrifugation at 1,560 *g* for 20 minutes at 20°C. After centrifugation, supernatant was pooled, and aliquots of 50 μL were frozen in liquid nitrogen and stored at −80°C.

**Spiking experiment**

PC3 EV and plasma samples were thawed on melting ice. Plasma was diluted 10-fold in citrate PBS (154 mmol/L NaCl, 1.24 mmol/L Na_2_HPO_4_.2H_2_O, 0.2 mmol/L NaH_2_PO_4_.2H_2_O, pH 7.4; 50 nm filtered (Nuclepore, GE Healthcare), supplemented with 0.32% trisodium citrate). Volumetric dilutions of 1:2, 1:5, 1:10, 1:50, 1:100 and 1:1000 of PC3 EVs in the diluted plasma was performed. 20 µL of plasma spiked with PC3 EVs was stained with 4 µL of CD63-PE (7.5 µg/mL, clone CLBGran/12, Beckman Coulter, Brea, CA), 4 µL of CD61-FITC (25 µg/mL, clone Y2/51, Dako, Glostrup, Denmark), 4 µL of CD235a-FITC (50 µg/mL, lot 83040, Dako, Glostrup, Denmark), 4 µL of CD45-FITC (50 µg/mL, clone HI30, Biolegend, San Diego, CA), and 4 µL of citrate PBS. Samples were incubated for 60 minutes at room temperature in the dark. The staining was stopped by addition of 200 µL citrate PBS.

All samples were analyzed for four minutes on an A50-Micro (Apogee, Northwood, UK) using side scatter triggering, at a flow rate of 4.5 µL/minute. PE signals were compensated for signal spill over from the FITC channel, and a gate identifying CD63-PE positive signals was set at 49 arbitrary units. The number of measured CD63-PE+ particles per dilution were determined, and compared to the 95% confidence interval determined using the number of CD63-PE+ particles found in plasma, assuming Poisson statistics. The minimum required concentration was determined by calculating the concentration of CD63-PE+ PC3 EVs present in the sample at the minimal volumetric dilution, using the concentration of CD63-PE+ particles in the original PC3 EV sample.
