# Supplementary material for: Detection of extracellular vesicles in plasma and urine of prostate cancer patients by flow cytometry and surface plasmon resonance imaging
Source: PLoS One. 2020 Jun 4;15(6):e0233443. doi: 10.1371/journal.pone.0233443 (PMC7272016; doi:10.1371/journal.pone.0233443)
Supplement: S1 Appendix — (DOC) [file pone.0233443.s002.doc]

**MIFlowCyt list**

| **Requirement** | **Please Include Requested Information** |
| --- | --- |
| 1.1. Purpose | In this study, we determined whether metastatic castration resistant prostate cancer (mCRPC) patients can be discriminated from healthy controls based on the presence of EV subtypes in plasma and/or urine samples using flow cytometry (FCM) and surface plasmon resonance imaging (SPRi). |
| 1.2. Keywords | biomarkers, exosomes, extracellular vesicles, flow cytometry, surface plasmon resonance |
| 1.3. Experiment variables | Sample type (i.e. patient or healthy control and urine or plasma), marker, technique (FCM or SPRi) |
| 1.4. Organization name and address | Amsterdam UMC – location AMC  Meibergdreef 9  1105 AZ Amsterdam  The Netherlands |
| 1.5. Primary contact name and email address | Linda Rikkert, l.g.rikkert@amsterdamumc.nl |
| 1.6. Date or time period of experiment | July 2019 – August 2019 |
| 1.7. Conclusions | Only the lactadherin^+^ particle and EV concentration in plasma measured by FCM differed significantly between patients and controls (p = 0.017). All other markers did not result in signals exceeding the background on both FCM and SPRi, or did not differ significantly between patients and controls. |
| 1.8. Quality control measures | Mean fluorescent intensity (MFI) was converted to molecules of equivalent soluble fluorochrome (MESF) for phycoerythin (PE) and fluorescein isothiocyanate (FITC) using the SPHERO PE Calibration kit (ECFP-F2-5K, Spherotech) and Quantum FITC-5 MESF beads (555A, Bangs Laboratories) respectively. Scatter signals were converted to scattering cross sections in nm^2^ with the use of Rosetta Calibration (Exometry, Amsterdam, The Netherlands). |
| 1.9 Other relevant experiment information | N/A |
| 2.1.1.1. (2.1.2.1., 2.1.3.1.) Sample description | Plasma and urine from mCRPC patients (n=5), and healthy controls (n=5). |
| 2.1.1.2. Biological sample source description | See Table 1 of the manuscript |
| 2.1.1.3. Biological sample source organism description | See Table 1 of the manuscript |
| 2.1.2.2. Environmental sample location | N/A |
| 2.2 Sample characteristics | Plasma is expected to contain EVs, lipoproteins and proteins. Urine is expected to contain EVs, salts and proteins. |
| 2.3. Sample treatment description | See ‘Sample collection’ in the Methods section of the manuscript. |
| 2.4. Fluorescence reagent(s) description | See Table 2 of the manuscript |
| 3.1. Instrument manufacturer | Apogee, Northwood, UK |
| 3.2. Instrument model | A60-Micro |
| 3.3. Instrument configuration and settings | Samples were analyzed at a flow rate of 3.01 μL/min on an A60-Micro, equipped with a 405 nm laser (100 mW), 488 nm laser (100 mW) and 638 nm laser (75 mW). Samples were measured for 4 minutes or until 500,000 events were detected while triggering on 405-nm side scatter using a threshold of 14 a.u., which corresponds to a side scattering cross section of 10 nm^2^ (Rosetta Calibration; Exometry, Amsterdam, The Netherlands). FITC fluorescence was collected in the 488-green channel (525/50 nm band pass filter), and PE fluorescence in the 488-orange channel (575/30 nm band pass filter). PMT voltages were set to 380 V for SALS, 350 V for LALS and 520 V for 488-green and 488-orange. |
| 4.1. List-mode data files | May be requested by emailing l.g.rikkert@amsterdamumc.nl |
| 4.2. Compensation description | No compensation was required since all samples were stained with a single fluorescent marker. |
| 4.3. Data transformation details | Data was transformed using MATLAB R2018b (Mathworks, Natick, MA) and FlowJo (v10.6.1; FlowJo, Ashland, OR).  Flow-SR was applied to determine the size and refractive index (RI) of particles and improve specificity by enabling label-free differentiation between EVs and lipoprotein particles (1, 2). Flow-SR was performed as previously described (1). Lookup tables were calculated for diameters ranging from 10 to 1000 nm, with step sizes of 1 nm, and RIs from 1.35 to 1.80 with step sizes of 0.001. The diameter and RI of each particle was added to the .fcs file by custom-build software (MATLAB R2018a). |
| 4.4.1. Gate description | First, for samples with a minimum count rate of 250 counts/s, time points of the data in which the count rate deviated >25% from the median count rate were removed to account for fluctuations in the flow rate. Next, fluorescent gates were set in such a way that (auto) fluorescence of the bulk of the events was excluded from the gate (Figure M1). Since there were slight variations in the level of bulk fluorescence on the different experiment days and between plasma and urine, gates were adapted based on the measurements of that day. Figure M1 shows a representative example of the gates used on the different experiment days. Lower bounds of the gates are listed in Table M1.  Because Flow-SR requires accurate measurement of both FSC and SSC, we applied Flow-SR only to particles with diameters >200 nm and fulfilling the condition:  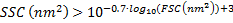 We subsequently discriminated between particles with an RI < 1.42, which are primarily EVs, and particles with an RI > 1.42. The fluorescent gates listed in Table M1 were applied to the RI < 1.42 and RI >1.42 populations. |
| 4.4.2. Gate statistics | Positive (^+^) events are defined as events with a fluorescent signal exceeding the gate. The number of positive events was corrected for flow rate, measurement time and dilutions performed during sample preparation. Median and standard deviation (SD) of the data was calculated per marker. The median is preferred over the mean, because the median is less influenced by outliers and therefore more representative given the small number of samples. Statistical analysis was performed using a Student’s t-test. All analyses were performed in MATLAB R2018b (Mathworks, Natick, MA). |
| 4.4.3. Gate boundaries | Table M1 shows the lower bounds of the gates per marker and experiment day. |

**References**

1. de Rond L, Libregts SFWM, Rikkert LG, Hau CM, van der Pol E, Nieuwland R, et al. Refractive index to evaluate staining specificity of extracellular vesicles by flow cytometry. J Extracell Vesicles. 2019;8(1):1643671.

2. van der Pol E, de Rond L, Coumans FAW, Gool EL, Böing AN, Sturk A, et al. Absolute sizing and label-free identification of extracellular vesicles by flow cytometry. Nanomedicine. 2018;14(3):801-10.

Table M1: Overview of the gates used per experiment day

| Experiment day | 1 | 2 | 3 | 4 |
| --- | --- | --- | --- | --- |
| Measured sample  (#patients/#healthy controls) | Plasma  (3/2) | Plasma  (2/3) | Urine  (3/2) | Urine  (2/3) |
| Marker↓ | Lower bound (MESF) | Lower bound (MESF) | Lower bound (MESF)) | Lower bound (MESF) |
| CD142 - PE | 85 | 90 | 85 | 80 |
| CD146 - FITC | 350 | 275 | 275 | 275 |
| CD235a - PE | 150 | 145 | 145 | 150 |
| CD24 - PE | 125 | 125 | 125 | 125 |
| CD45 - FITC | 630 | 570 | 325 | 400 |
| CD47 - PE | 105 | 75 | 75 | 75 |
| CD61 - PE | 85 | 75 | 75 | 75 |
| CD63 - PE | 85 | 75 | 75 | 75 |
| CD81 - PE | 75 | 75 | 75 | 75 |
| CD9 - PE | 77 | 75 | 75 | 75 |
| EpCAM - PE | 90 | 70 | 70 | 75 |
| Her2 - PE | 125 | 125 | 125 | 125 |
| Lactadherin - FITC | 375 | 375 | 300 | 300 |
| PSMA - PE | 105 | 75 | 75 | 80 |

MESF: Molecules of equivalent soluble fluorochrome


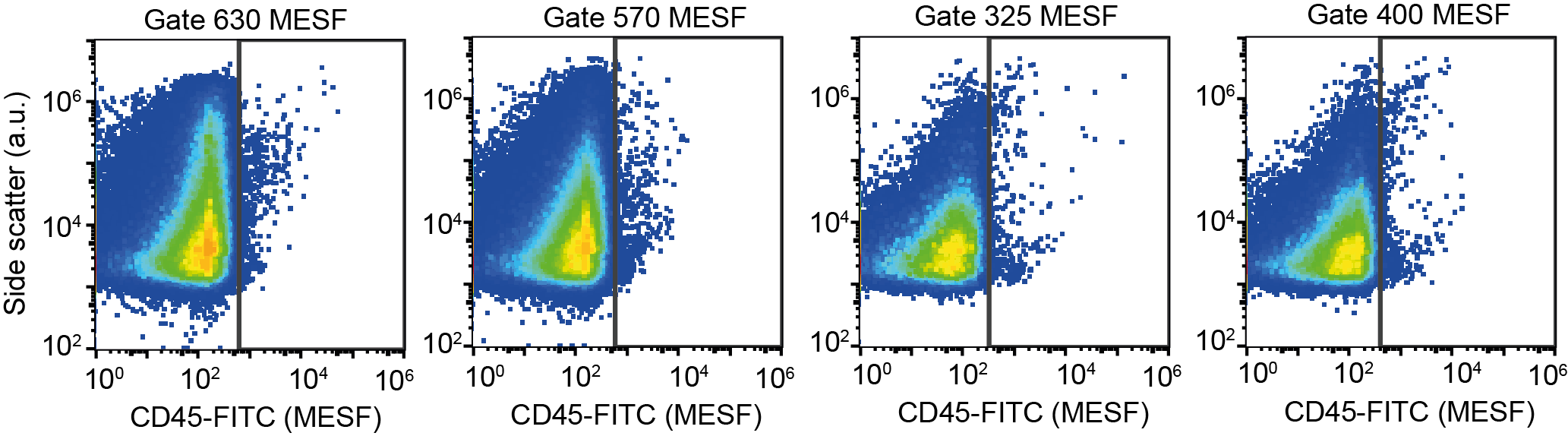


**Figure M1**: Representative example of the gates used on the experiment days. Shown are side scatter versus CD45-FITC fluorescence of samples measured on experiment day 1 (left graph) to 4 (right graph). MESF: Molecules of equivalent soluble fluorochrome.
